# HIV-associated Kaposi sarcoma: Advanced disease and high mortality of patients referred to a multidisciplinary clinic in South Africa, 2022–2024

**DOI:** 10.4102/sajhivmed.v27i1.1803

**Published:** 2026-04-24

**Authors:** Caitlin S. Turner, Tamsin Lovelock, Zahiera Ismail, Henriette Burger, Hans W. Prozesky, Jantjie Taljaard

**Affiliations:** 1 Division of Infectious Diseases, Department of Medicine, Faculty of Medicine and Health Sciences, Stellenbosch University, Cape Town, South Africa; 2 Division of Radiation Oncology, Department of Medical Imaging and Clinical Oncology, Faculty of Medicine and Health Sciences, Stellenbosch University, Cape Town, South Africa

**Keywords:** Kaposi sarcoma, HIV, HHV-8, visceral Kaposi sarcoma, HIV-associated malignancies, South Africa, TB coinfection, outcome

## Abstract

**Background:**

Kaposi sarcoma (KS) remains the most common HIV-associated malignancy in South Africa and continues to contribute substantially to the national disease burden, despite the widespread availability of antiretroviral therapy (ART) since 2004.

**Objectives:**

To describe the clinical characteristics and outcomes of patients referred to a multidisciplinary HIV-associated KS clinic.

**Method:**

A retrospective, descriptive cohort study was conducted at a South African tertiary hospital, including patients diagnosed with HIV-associated KS between January 2022 and December 2024. Data were deidentified, pooled, and analysed using descriptive statistics and univariate analysis.

**Results:**

A total of 137 patients were included. Most were on ART at the time of KS diagnosis, with a median CD4 count of 122 cells/µL. Advanced disease was common, with 85% of patients presenting with AIDS Clinical Trial Group (ACTG) poor risk tumour stage KS, including 43% with visceral involvement. The 1-year all-cause mortality rate was 38%, with visceral KS and lower CD4 counts on presentation being significantly associated with increased risk of death. HIV viral suppression was associated with improved survival, while duration of ART prior to KS diagnosis did not significantly affect outcomes.

**Conclusion:**

These findings highlight the late stage at presentation and poor prognosis of HIV-associated KS in this cohort, underscoring the urgent need for improved retention in HIV care and earlier diagnosis of KS.

**What this study adds:** This study of HIV-associated KS in the ART era, providing insights into clinical presentation, staging and factors associated with 1-year mortality.

## Introduction

Kaposi sarcoma (KS) is a vascular endothelial cell malignancy caused by human herpesvirus-8 (HHV-8), also referred to as KS-associated HHV (KSHV). Kaposi sarcoma has a well-established link with HIV and is the most strongly HIV-associated cancer among the AIDS-defining malignancies.^1^ Within the Southern African context, there is a high burden of KS disease as a result of high HIV prevalence as well as high HHV-8 seroprevalence.^2,3,4^ Globally, sub-Saharan Africa bears the highest incidence and mortality rates of KS.^2,3,4,5^ Despite widespread availability of antiretroviral therapy (ART) in South Africa, KS continues to represent a significant burden on the healthcare system.^6^ The persistence of KS, despite widespread ART access, is largely attributed to delayed HIV diagnosis and suboptimal adherence and retention in HIV care programmes.^7^ Previous studies have described HIV-associated KS in South Africa, but publications from the last 10 years are scarce.^8,9,10^

Our study is a retrospective cohort of HIV-associated KS, detailing clinical features, staging, immune status, and short-term outcomes two decades after national ART rollout.

## Research methods and design

Tygerberg Hospital is a tertiary care teaching hospital in Cape Town, South Africa. This retrospective descriptive cohort study took place within the Tygerberg Hospital Adult Infectious Diseases outpatient clinic (IDC), which acts as the HIV-associated KS referral clinic for the subregion. The Tygerberg Hospital IDC reviews up to 60 new cases of KS annually and conducts a weekly multidisciplinary KS conference. The clinical records of all adult patients who presented to Tygerberg Hospital IDC between 01 January 2022 and 31 December 2024, with a histologically or immunohistochemically confirmed diagnosis of HIV-associated KS, were reviewed.

Data collected included demographic information and KS-specific clinical information, including the AIDS Clinical Trial Group (ACTG) classification (Table 1). Information related to HIV and tuberculosis (TB) was also collected. Dates of death were collected from clinical records or the South African Medical Research Council National Population Register death registry for patients with South African identification numbers. For living patients, dates of last clinical contact with the provincial health system were collected from Western Cape electronic health records databases.

**TABLE 1:** Adapted AIDS Clinical Trial Group classification for HIV-associated Kaposi sarcoma.^8,11,12,13^

Type of risk	Tumour stage (T)	Immune status (I)	Severity of systemic infection (S)
Good risk (subscript 0 and all the following)	Minimal involvement of mucosa and/or isolated skin involvement and/or isolated nodal disease	CD4 cell count ≥ 150 cells/μL	No history of opportunistic infections or candidiasis, Karnofsky performance status > 70%, no constitutional ‘B’ symptoms (night sweats, significant loss of weight, unexplained fever, diarrhoea > 2 weeks)
Poor risk (subscript 1 and any of the following)	Extensive mucosal involvement or any visceral involvement or associated lymphoedema or ulceration	CD4 cell count < 150 cells/μL	Presence of opportunistic infections (including tuberculosis) and/or candidiasis or ‘B’ symptoms noted or poor performance status (Karnofsky performance status < 70%).

Note: Please see the full reference list of the article, Turner CS, Lovelock T, Ismail Z, Burger H, Prozesky HW, Taljaard J. HIV-associated Kaposi sarcoma: Advanced disease and high mortality of patients referred to a multidisciplinary clinic in South Africa, 2022–2024. S Afr J HIV Med. 2026;27(1), a1803. https://doi.org/10.4102/sajhivmed.v27i1.1803, for more information.

ACTG, AIDS Clinical Trial Group; KS, Kaposi sarcoma.

Survival outcome was determined at 1 year from first visit to the IDC. Outcomes were categorised as deceased, alive or not in care. Patients were classified as deceased if a date of death (DOD) on or before 1 year was recorded. Patients were classified as alive at 1 year if their recorded DOD was longer than 1 year from presentation or if they had documented contact with provincial health services on or after 1 year from presentation. All other patients were classified as not in care and were excluded from 1-year overall survival analysis.

All data were captured using a REDCap database and exported into Microsoft Excel for data cleaning and analysis. Data variables were described with descriptive statistics and analysis performed using Microsoft Excel’s inbuilt features as well as Python (version 3.14; Python Software Foundation, Beaverton, Oregon, United States) in Jupyter Notebook (version 7.5.4; Project Jupyter, IPython Project) with the pandas package for data handling and lifelines for survival analysis. Statistical significance of clinical associations with living status was calculated using chi-square analysis and Fisher’s exact test for groups smaller than five. Survival was analysed using Kaplan Meier curves with 95% confidence intervals included. The crude surviving proportion was calculated at 1 year from presentation and reported as the primary outcome. Welch T Tests were utilised for comparison of continuous variables. A *P*-value < 0.05 was considered statistically significant.

### Ethical considerations

Ethical clearance to conduct this study was obtained from the Stellenbosch University Research Ethics Committee (No. U25/05/391). Hospital approval was obtained from Tygerberg Hospital National Health Research Database (NHRD) (reference number WC_202503_009). All data were deidentified and stored securely to maintain confidentiality.

## Results

A total of 137 patients presented to the Tygerberg Hospital IDC with a diagnosis of HIV-associated KS between 01 January 2022 and 31 December 2024 (Table 2).

**TABLE 2:** Demographic, disease and treatment characteristics (*N* = 137).

Variable	*n*	%	Median	Range
Age (years)	-	-	39	16–68
**Gender**				
Male	81	59	-	-
Female	56	41	-	-
Baseline CD4 count (cells/μL)	-	-	122	1–759
**KS stage**				
T-stage:				
0	20	15	-	-
1	117	85	-	-
I-stage:				
0	57	42	-	-
1	80	58	-	-
S-stage:				
0	60	44	-	-
1	77	56	-	-
Good Risk (all subscript 0)	6	4	-	-
Poor Risk (any subscript 1)	131	96	-	-
T1S1	69	50	-	-
Non-T1S1	68	50	-	-
On ART at time of diagnosis	117	85	-	-
**Treatment (multiple modalities possible)**				
ART	137	100	-	-
Chemotherapy	96	70	-	-
Radiotherapy	4	3	-	-
**Outcomes at 1 year**				
Confirmed deceased	48	35	-	-
Confirmed alive	80	58	-	-
Not in care	9	7	-	-

ART, Antiretroviral treatment; KS, Kaposi sarcoma; T1S1, tumour stage 1, systemic involvement stage 1.

The median age at presentation was 39 years and the cohort was predominantly male (59%). Most patients (85%) were taking ART at the time of KS diagnosis and the median duration of ART prior to presentation to the clinic was 38 months (range: 0 months – 242 months). The median CD4 count was 122 cells/μL (range: 1 cells/μL – 759 cells/μL). Almost all patients (99%) had histologically confirmed KS, and 96% of samples were positive for HHV8. The most commonly biopsied site was the skin (92%), followed by the gastro-intestinal tract (GIT), mucosal sites and lymph nodes.

There were 36 patients with a CD4 count of > 200 cells/μL. Thirty-five of these patients presented with cutaneous involvement. The remaining patient had isolated mucosal involvement. Three of these patients had conjunctival involvement, one had perianal, and another had penile involvement. None had confirmed nodal involvement. Ten had visceral involvement, four of whom had biopsy confirmed gastrointestinal KS. Four had pulmonary KS. One patient had extensive visceral involvement with both the lung and GIT involved. Two patients had atypical visceral involvement – one with pericardial involvement and the other with a spinal lytic lesion.

Fifty-two patients (38%) were taking TB treatment at the time of presentation. Microbiological confirmation of TB was documented in 75% of these patients. Of the remaining 13 patients receiving empiric TB treatment, four were later diagnosed with pulmonary KS.

Skin involvement was the predominant clinical feature, present in 94% of patients, with lesions affecting the lower limb observed in 73%, of whom 61% had lymphoedema. Mucosal involvement was present in 66% of patients, most commonly affecting oral mucosal surfaces and predominantly the hard palate. Non-oral mucosal sites such as the conjunctiva and genitalia were less common (Table 3).

**TABLE 3:** Clinical characteristics.

Variable	*n*	%
**Cutaneous involvement†**	129	94
Lower limbs	100	73
Upper limbs	65	47
Trunk	52	38
Head and/or neck	48	35
**Total mucosal involvement:†**	90	70
Oral – total†	85	63
Hard palate	72	53
Tongue	6	4
Tonsils	5	4
Oropharynx	6	4
Soft palate	2	1
Lip	2	1
Gingiva	2	1
Not otherwise specified	7	5
Other mucosal sites – total:†	13	9
Conjunctival	8	6
Vulval	3	2
Perianal	2	1
Penile	1	1
Nodal involvement	5	4
Lymphoedema	75	55
**Visceral involvement†**	59	43
Pulmonary (all):	41	30
Pulmonary only	24	18
Pulmonary – Bronchoscopy confirmed	1	2
GIT (all):	31	23
GIT only	14	12
GIT – Biopsy confirmed	10	32
Pulmonary and GIT	16	12
Pericardial	4	3
Bone and/or spine	3	2

GIT, gastrointestinal.

† , Multiple sites possible.

Nineteen patients underwent diagnostic fine needle aspiration (FNA) or biopsy because of significant lymphadenopathy. Only five patients (4%) had confirmed KS lymphadenopathy.

Visceral involvement was identified in 59 patients (43%). The most common sites affected were the lung and the lung and gastrointestinal system (GIT). Less common visceral locations included the pericardium (*n* = 4) and bone/vertebrae (*n* = 3). A CD4 count of < 200 cells/µL at initial presentation was significantly associated with visceral involvement (*P* = 0.03).

Pulmonary involvement was confirmed by bronchoscopy in only one patient; the remainder were diagnosed based on findings from chest radiography or CT imaging. Among those with pulmonary KS, 39% had a pleural effusion.

Most patients with visceral involvement also had mucosal involvement (76%). Visceral involvement was not seen in the absence of mucosal or cutaneous KS; however, mucosal involvement was seen without cutaneous involvement (*n* = 8).

All patients received ART, 96 patients (70%) were treated with chemotherapy and four received radiotherapy. Standard chemotherapy regimens used at Tygerberg Hospital are six to eight intravenous cycles of bleomycin and vincristine, or single-agent paclitaxel. We were unable to report on the outcomes of the different treatment modalities as it is the subject of another ongoing study.

**FIGURE 1:**
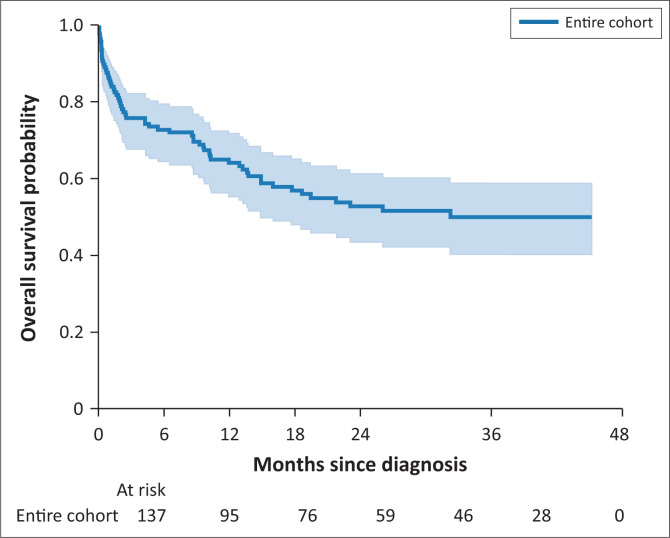
Survival curve, entire cohort.

At 1 year after first presentation to the IDC, 48 patients were confirmed to be deceased, 80 were confirmed to be alive and nine were not in care. The percentage of patients alive at 1 year (excluding patients not in care) was 62.5%. Figure 1 depicts overall survival probability.

Poor-risk KS classification, lower CD4 count on presentation, and lack of viral suppression were associated with increased mortality (Table 5). Median CD4 count on presentation was 137 cells/μL for patients who were alive at 1 year, in comparison to 67 cells/μL for those who were deceased (*P* = 0.004). Mortality was higher among patients with a CD4 count of less than 100 cells/μL than those with a CD4 count of less than 150 cells/μL. An unsuppressed HIV viral load (VL) was also associated with increased mortality (*P* = 0.003). Among the 100 people with follow-up HIV VL data available, only 69% of patients were virally suppressed after KS diagnosis. Of the virally suppressed patients, 12% were deceased at 1 year, compared to 42% of the virally unsuppressed patients. Surprisingly, mortality among those who had been on ART for more than 12 months prior to KS diagnosis was similar to that of patients who had been on ART for less than 12 months and there was no association between patients receiving ART before diagnosis and death (*P* = 0.33). However, mortality was still high (47%) among those who were not on ART at the time of diagnosis.

Co-infection with TB was also common with KS, with more than one-third of patients being on TB treatment at the time of KS diagnosis (Table 4). There were 14 patients who had a diagnosis of TB at the time of KS diagnosis and were later diagnosed with pulmonary KS. Of those patients, four were on empiric treatment for TB (no laboratory confirmation of diagnosis) and 10 had confirmed TB. Those who had confirmed TB comorbid with pulmonary KS had a 1-year mortality of 71%. Laboratory results relating to pleural effusions in patients with pulmonary KS and TB were documented and both a higher adenosine deaminase (ADA) and lactate dehydrogenase (LDH) were noted in the TB group compared to the pulmonary KS group.

**TABLE 4:** Tuberculosis characteristics.

Variable	*n*	%	Median	Range
Previous TB diagnosis	54	39	-	-
**Receiving TB treatment at time of KS diagnosis**	52	38	-	-
Treatment for laboratory-confirmed TB	39	75	-	-
Empiric TB treatment	13	25	-	-
**Any pleural effusion**	21	15	-	-
Pulmonary KS with a pleural effusion:	16	-	-	-
ADA (U/L) (*n* = 11)	-	-	10.4	2.5–30.1
LDH (U/L) (*n* = 12)	-	-	150	42–433
TB with pleural effusion:†	5	-	-	-
ADA (U/L) (*n* = 5)	-	-	28.3	15.5–47.6
LDH (U/L) (*n* = 5)	-	-	705	175–832
TB diagnosis at any point after KS diagnosis	23	17	-	-

TB, tuberculosis; KS, Kaposi sarcoma; ADA, adenosine deaminase; LDH, lactate dehydrogenase.

† , One patient was on empiric TB treatment, three patients had laboratory-confirmed TB, and the remaining patient had a positive urine lipoaribinomannan (ULAM) test.

Visceral KS involvement was significantly associated with a poor prognosis (Table 5). A large percentage of those with probable pulmonary involvement (54%) were deceased at 1 year, with a 90-day mortality rate of 36%. When looking at the GIT group, more than half (63%) of this group were deceased at 1 year, and there was a 90-day mortality of 50%. Dual pulmonary and GIT involvement was associated with an 69% mortality at 1 year and a 54% 90-day mortality.

**TABLE 5:** Mortality by risk factors (*N* = 128).

Variable	90 days		1 year	
	%	*P*	%	*P*
Total	25.78		37.5	
**Tumour stage 1**		< 0.001		< 0.001
T1S1	38.46	-	56.92	-
Non-T1S1	12.70	-	17.46	-
**Visceral involvement**	-	< 0.001	-	< 0.001
Any	40.35	-	56.14	-
None	13.89	-	22.22	-
**CD4: 100 cells/μL**	-	0.004	-	0.02
<	37.93	-	48.28	-
>	15.71	-	28.57	-
**CD4: 150 cells/μL**	-	0.03	-	0.09
<	32.89	-	43.42	-
>	15.38	-	28.57	-
**CD4: 200 cells/μL**	-	0.06	-	0.049
<	29.90	-	42.27	-
>	12.90	-	22.58	-

T1S1, tumour stage 1, systemic involvement stage 1; Non-T1S1, non-tumour stage 1, non-systemic involvement stage 1.

**FIGURE 2:**
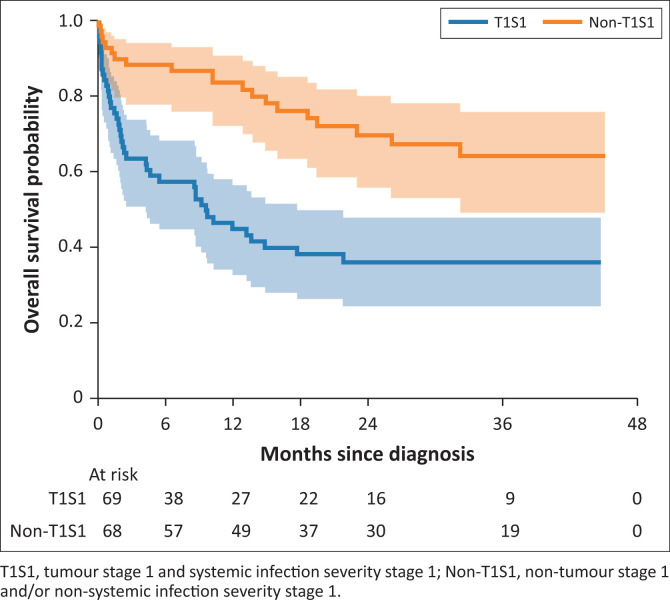
One-year overall survival according to T1S1 and non-T1S1 groups.

Most patients had advanced disease at the time of presentation, with 85% of patients presenting with tumour stage 1 (T1) disease, and 50% with tumour stage 1 as well as severity of systemic infection stage 1 (T1S1) disease. These were all associated with increased mortality (Table 5). Comparing the T1S1 group with the non-T1S1 group showed 1-year mortality of 57% in the former, and 17% in the latter. Median overall survival of the T1S1 group was 293 days (Figure 2) and was not reached in the non-T1S1 group.

## Discussion

People with HIV-associated KS present with a wide spectrum of clinical manifestations. In this cohort, 94% had cutaneous involvement, predominantly affecting the lower limbs, and 66% had mucosal involvement. This correlates with previous research which details lower limb cutaneous presentation as the most common site.^8,14^ Interestingly, 6% of patients did not have any cutaneous involvement – a presentation which is thought to be rare, although it is well described.^15,16^ Mucosal involvement predominantly occurred in the oral cavity, with less frequent involvement of sites such as the conjunctiva and genitalia. These findings underscore the importance of including KS in the differential diagnosis of mucocutaneous lesions in people with HIV, even when presentations are atypical.

A large proportion of patients in this study presented with poor risk HIV-associated KS, and subsequent poor clinical outcomes. A median period of 65 days from first clinic presentation to death also suggests that late presentation resulted in limited treatment benefit and high mortality. Visceral involvement was noted in a large subset of patients and was associated with statistically significant mortality. Visceral involvement is thought to be a result of disease progression and may be an indicator that patients are identified or referred late. Visceral involvement may be challenging to diagnose in patients with advanced HIV disease because of non-specific symptoms and limited primary care resources available for diagnosis.^17^ However, in our cohort, all patients with visceral involvement also had either skin or mucosal lesions on presentation.

Gastrointestinal involvement was seen in 23% of patients and was diagnosed via gastroscopy or gastric biopsy. Mortality associated with gastrointestinal KS was notably higher than that of pulmonary KS. This may reflect a referral bias, as chest radiographs were routinely performed for T1 patients, those with pulmonary symptoms, and suspected TB cases, potentially enabling earlier identification of pulmonary KS. Conversely, gastrointestinal KS was likely under-recognised, with only patients exhibiting gastrointestinal symptoms or unexplained anaemia undergoing endoscopic evaluation.

The poor risk classification at presentation (96%) reported in this study appears to be a common thread among studies conducted in sub-Saharan Africa.^8,18^ Data obtained from the same IDC at Tygerberg Hospital in 2014 yielded similar results, with 91% having poor risk disease.^8^ Since the widespread rollout of ART in 2004, it was anticipated that the incidence and mortality of HIV-associated KS would decline. This was supported by earlier studies, which saw a reduction in mortality from KS amongst patients who presented between 2009 and 2012 in comparison to patients presenting between 2004 to 2008, coinciding with the national rollout.^9^ However, the findings from this study suggest that this reduction in mortality may not have been sustained. Other studies published within South Africa, dating back more than 10 years, show a 1-year overall survival of between 60% to 80%.^9,10,19^ Our 1-year overall survival of 62.5% is on the lower end of this range, despite improved access to ART and higher baseline median CD4 counts.

Tuberculosis continues to pose a significant public health challenge in the Western Cape, South Africa, where an estimated 160 000 cases were diagnosed over the course of the study period.^20^ In high TB burden settings, empiric treatment is frequently initiated based on clinical suspicion using symptoms and chest radiograph findings, even when laboratory results are negative.^21^ In this cohort, 75% of patients receiving TB treatment at the time of KS diagnosis had laboratory-confirmed TB. Patients with pulmonary KS may present with symptoms and chest radiograph findings which mimic pulmonary TB, so while some of these patients likely had true TB co-infection, a subset may have had pulmonary KS which was misdiagnosed as TB, delaying appropriate referral and management.^21^ Fourteen patients were initially treated for TB and later diagnosed with pulmonary KS. Four of these patients did not have a laboratory confirmation of TB and were receiving empiric TB treatment. It is important to note that most diagnoses of pulmonary KS in this study were presumptive and based on typical chest radiograph findings. Pleural effusion laboratory results were not sufficient to draw any conclusions. Within a resource-limited setting with a high burden on specialised services, advanced imaging and invasive diagnostic methods are often not readily available, further increasing the risk for incorrect or delayed diagnosis.

Several factors in the South African context hinder effective HIV care and contribute to the high prevalence of advanced KS. Prior qualitative research has highlighted patient-level barriers to HIV treatment such as poor disease awareness, limited personal agency, lack of social support, and the use of traditional medicine, all of which may delay diagnosis and treatment. Health system-related barriers were numerous and included delayed referrals, misdiagnoses, inadequate physical examinations, and limited access to essential diagnostic tools such as tissue biopsy equipment.^7^ Many of these factors were likely also present within the context of this study.

A positive finding is that 85% of this cohort were already on ART at the time of their diagnosis. Although this doesn’t reach the Joint United Nations Programme on HIV/AIDS (UNAIDS) 95% goal for 2030, it is a positive step forwards, especially when compared to the 2014 cohort from the same hospital (85% vs 67%).^8,22^ Unfortunately, viral suppression was only achieved in 69% of patients, with this being a significant protective factor against death. Patients presenting with CD4 counts < 100 cells/µL experienced higher mortality. Although not unexpected, these findings underscore the urgent need to retain our patients in care after HIV diagnosis to maintain viral suppression, achieve durable immune reconstitution and prevent sequalae of disease such as KS.

### Limitations

Because of the retrospective design, there is inherent limitation and bias in utilising data that may be unreliable or incomplete. The clinic is situated within a tertiary referral hospital, with a potential referral bias for more severe disease. The brief follow-up period of this study makes it impossible to comment on longer-term outcomes and survival. Vital status could not be confirmed for nine patients; eight of whom were foreign nationals and one patient who was an incarcerated individual, potentially underestimating mortality. It was also not possible to comment on the cause of death. This study did not look at the effect of oncological treatment on outcomes because of ongoing research elsewhere.

## Conclusion

Despite widespread access to ART in South Africa, patients with HIV-associated KS continue to present to tertiary care facilities with advanced disease, resulting in high mortality rates, even in settings with specialist care. Visceral involvement was common and predicted poor outcomes. These findings highlight the importance of considering visceral KS in people with HIV, particularly in high TB-burden settings where diagnostic overlap may contribute to delayed or missed diagnoses.
